# The visibility of smoking in Europe and its relationship with youth’s positive beliefs about smoking

**DOI:** 10.1007/s00038-019-01288-z

**Published:** 2019-09-05

**Authors:** Naomi A. Lagerweij, Mirte A. G. Kuipers, Michael Schreuders, Adeline Grard, Martin Mlinarić, Matthias Richter, Teresa Leão, Jaana M. Kinnunen, Anton E. Kunst

**Affiliations:** 1 Department of Public Health, Amsterdam Public Health Research Institute, Amsterdam UMC, University of Amsterdam, Amsterdam, The Netherlands; 2 Institute of Health and Society (IRSS), Université catholique de Louvain, Brussels, Belgium; 3 Institute of Medical Sociology (IMS), Medical Faculty, Martin Luther University Halle-Wittenberg, Halle (Saale), Germany; 4 National School of Public Health, NOVA University of Lisbon, Lisbon, Portugal; 5 Faculty of Social Sciences, Health Sciences, Tampere University, Tampere, Finland; 6 Trimbos Institute, Utrecht, The Netherlands

**Keywords:** Smoking, Perception, Awareness, Adolescent, Europe, Smoke-free policy

## Abstract

**Objectives:**

To determine adolescent-reported visibility of smoking in different public and private spaces in Europe and associations between smoking visibility and beliefs about the benefits of smoking.

**Methods:**

We used SILNE-R cross-sectional survey data (2016/2017) of 10,798 14–16-year-old students from 55 secondary schools in seven European cities. Respondents reported for private and public spaces whether they had seen others smoke there in the last 6 months. Beliefs about the benefits of smoking were measured on a 7-item scale; higher scores indicated more positive beliefs. Multilevel linear regression analyses determined associations while controlling for potential confounders and stratifying by smoking status.

**Results:**

Most students reported observing others smoke in public spaces, especially at train/bus stations (84%). Positive beliefs about smoking of never smokers were positively associated with seeing others smoke in train/bus stations and leisure/sports facilities, but not at home, a friend’s home, restaurants or bars, when fully adjusted. Associations were of similar magnitude for ever smokers.

**Conclusions:**

Smoking in several public places is highly visible to adolescents. Reducing this visibility might weaken positive beliefs that adolescents have about smoking.

**Electronic supplementary material:**

The online version of this article (10.1007/s00038-019-01288-z) contains supplementary material, which is available to authorized users.

## Introduction

Over the last two decades, smoke-free policies have been implemented widely throughout Europe, contributing to a reduced occurrence of smoking in different public spaces (Nagelhout et al. 2011; Sureda et al. 2014; Van Beek et al. 2018). Comprehensive smoke-free policies have the potential to reduce the social acceptability of smoking (Alesci et al. 2003; Albers et al. 2004), as well as the smoking prevalence and smoking uptake (Song et al. 2015; Wakefield et al. 2000) among youth. However, in several European countries smoking bans are partially implemented or poorly enforced in indoor spaces, and smoking is still allowed in most outdoor public spaces (Joossens and Raw 2017), thus maintaining the visibility of smoking and slowing down its denormalization. For example, in a recent European study on 16 cities in 8 European countries, smoking was observed in 13% of bars where smoking was banned and in 90% of bars where smoking was legally allowed (Van Beek et al. 2018). In addition, the visibility of smoking may continue to be high in private spaces, such as family homes: a 2009 survey found that 38% of European adults allowed smoking in the home (TNS Opinion and Social 2010).

The visibility of smoking has mostly been studied from the perspective of adults, and not adolescents, while seeing others smoke may shape youth beliefs about smoking and may lead to youth imitating others’ smoking behaviour (Bandura 1986). Smoking beliefs can be defined as a range of individual attitudes and opinions a person holds about tobacco smoking, including beliefs about the negative health consequences and social benefits of smoking. Beliefs about the social benefits of smoking possibly have a greater effect on adolescents’ smoking intentions and behaviour than knowledge of the negative health consequences of smoking (Dalton et al. 1999; Halpern-Felsher et al. 2004), indicating the importance of studying the relationship between such beliefs and the visibility of smoking. For example, adolescents may believe that smoking helps to relax or that it makes you look more mature or attractive. One study found that observing people smoking in movies was associated with an increased number of positive beliefs about the social benefits of smoking among adolescents (Sargent et al. 2002). To our knowledge, this association has not been studied for the visibility of smoking in the various real-life settings adolescents encounter.

We hypothesize that the influence of the visibility of smoking on positive beliefs about smoking depends on the location of exposure. Firstly, observing others smoke in everyday-life contexts where smoking is not common, such as restaurants, may contribute to more positive opinions of smoking (Alesci et al. 2003) than observing others smoke while going out in bars and clubs, as smoking at these places is already common and a socially acceptable behaviour (Nichter et al. 2010; Rooke et al. 2013).

Secondly, the smoking beliefs of adolescents may be influenced by the type of people they observe smoking in specific locations, depending on the extent to which these people are role models. In private spaces (i.e. own or friends’ homes), adolescents may especially observe smoking by peers and parents smoke, who are typical role models that may influence adolescents’ smoking beliefs (Rodriguez et al. 2007) and smoking status (Alves et al. 2016). In this line of reasoning, public spaces such as sports facilities may also be particularly influential because there youth may watch smoking by peers, parents, and sports teachers. The influence of public spaces where adolescents are more likely to observe smoking of strangers (e.g. at train stations) has not been previously studied, but may be expected to be weaker as such people may be less likely to function as role models.

The relationship between seeing others smoke and positive beliefs may also differ according to the individual smoking status of the adolescent. We hypothesize that adolescents who have experienced smoking (i.e. ever smokers) have already formed ideas about the benefits of smoking based on their previous experiences. In addition, in accordance with cognitive dissonance theory, smokers rationalize and maintain their smoking behaviour by endorsing positive beliefs about smoking (Fotuhi et al. 2013). The visibility of smoking may, therefore, not further influence the beliefs of ever smokers, but only of those who have never experienced smoking before (i.e. never smokers).

This European study had three aims: (1) to assess the adolescent-reported visibility of smoking in different public and private spaces, (2) to determine whether the visibility of smoking in these spaces was associated with positive beliefs about smoking, and (3) to analyse these associations according to smoking status.

## Methods

### Design and study population

Our study used existing data from an international survey carried out as part of the European SILNE-R project. Data were collected by means of a paper-based survey on tobacco use between late 2016 and late 2017. The surveys were completed in the classroom under surveillance of a research assistant and teachers by 13,061 students in 55 secondary schools in seven medium-sized European cities with a socio-economic context similar to the national average. The cities were: Dublin (Ireland), Tampere (Finland), Amersfoort (The Netherlands), Namur (Belgium), Latina (Italy), Hannover (Germany), and Coimbra (Portugal). Schools in the participating cities were selected from a variety of neighbourhoods and represented different educational levels. In each school, two grades that enrolled 14-to-16-year-old students were selected, resulting in an age range of 12–19. Ethical approval for the study was obtained separately in each country. The survey was a partial replication of a previous survey, detailed in Lorant et al. (2015).

The overall participation rate was 79.9%. The participation rates in the seven cities were: 81.1% in Dublin, 87.9% in Tampere, 85.8% in Amersfoort, 85.4% in Belgium, 82.2% in Italy, 62.0% in Germany, and 76.3% in Portugal. For the analyses, only 14-to-16-year-olds (*N *= 11,381) were included to represent our target group. In addition, we excluded individuals with missing information on gender (*N *= 15), migration background (*N *= 192), respondents’ smoking status (*N *= 48), best friends’ smoking status (*N *= 54), smoking beliefs (*N *= 60), and the visibility of smoking at home (*N *= 49), a friend’s home (*N *= 31), bars/clubs (*N *= 44), restaurants (*N *= 40), a train or bus station (*N *= 44), and leisure/sports facilities (*N *= 28), resulting in a study population of *N *= 10,798.

### Measures

#### Dependent variable

The dependent variable was ‘positive beliefs about smoking’. On a four-point Likert scale, respondents indicated whether they ‘completely disagreed’, ‘disagreed’, ‘agreed’, or ‘completely agreed’ with the following seven statements: ‘Smoking increases your chances of (1) looking cool, (2) feeling relaxed, (3) becoming popular, (4) looking grown-up, (5) losing weight or keeping thin, (6) appearing sexy/attractive, (7) getting a boyfriend/girlfriend’. Four of these items were based on the social beliefs mentioned in Song et al. (2009). The Cronbach’s alpha for the seven items was *α* = 0.81. Respondents received 0 to 3 points on each item (0 for ‘completely disagree’ and 3 for ‘completely agree’). For each individual, the mean of all seven items resulted in the positive beliefs score, ranging from 0 to 3. Missing values on an item were replaced with the mean of the remaining items. Respondents with more than two missing values on the seven items were excluded. Higher scores indicated more positive beliefs about smoking.

#### Independent variable

The main independent variable was the reported visibility of smoking at six different spaces: home, friend’s home, bars/cafes/clubs/discos, restaurants (including fast food/diners), train or bus station, and leisure/sports facilities. Respondents had to indicate whether they had seen people smoke in these locations within the last 6 months. The response categories were ‘yes’, ‘no’, and ‘I never go to these places’. The survey question did not explicitly distinguish between smoking inside or just outside bars/clubs and restaurants.

#### Other covariates

Socio-demographic variables such as age, gender, migration background, and parental educational level were included as potential confounders as they may be related to beliefs and norms about (Lee et al. 2013; Taylor et al. 1999; Urberg and Robbins 1981; Wilkinson et al. 2009), and to exposure towards other smokers (Whitlock et al. 1998). For migration background, respondents were categorized as having zero, one, or two parents born in a country other than the country of residence. As an indicator of socio-economic status, we measured the level of education of the most highly educated parent (i.e. information of either the mother or the father was used). The parental educational level was measured on a country-specific scale and categorized into ‘low’, ‘middle’, ‘high’, and ‘unknown’. In general, ‘low’ was equivalent to no schooling, primary school, and/or lower level of secondary school, ‘middle’ was equivalent to completed secondary school and/or lower level of college, and ‘high’ was equivalent to a college or university degree.

The variable ‘country’ was included as a covariate, as there are differences between countries in the legislative comprehensiveness of smoke-free policies (Joossens and Raw 2017), the legal age to buy tobacco products, and cultural norms, which may all influence the visibility of and positive beliefs about smoking.

Visibility of smoking and positive beliefs about smoking are both likely to be influenced by the smoking status of respondents, friends, and parents (Halpern-Felsher et al. 2004; Urberg and Robbins 1981; Wilkinson et al. 2008). Respondents were categorized into ever smokers and never smokers. ‘Ever smokers’ were defined as those who had ever tried cigarette smoking, even if it was just a few puffs. ‘Never smokers’ had never tried cigarette smoking, not even one puff. Regarding the smoking status of friends, respondents were asked whether any of their best and closest friends smoke cigarettes and were categorized into ‘none’, ‘some’, ‘most’, and ‘all’. For ‘parental smoking status’, respondents reported whether their parents and/or stepparents currently smoke or do not smoke (including ex-smokers). We categorized the number of smoking parents into ‘none’, ‘one’, and ‘two or more’.

### Statistical analysis

Descriptive statistics of the study population were provided, stratified by smoking status. To account for the hierarchical data structure (students within schools within cities), multilevel analyses were performed. Due to a limited number of schools within cities, we could only perform a two-level model (students within schools). Multilevel linear regression analyses determined the association between reported visibility of smoking in each location and positive beliefs about smoking. Model 0 was a crude model. Model 1 included adjusting for age, gender, migration, parental education, and country. Model 2 additionally included the smoking status of the respondents, best friends, and parents. Model 3 additionally included the reported visibility of smoking in the five other locations. The analyses were stratified by smoking status of the respondents. Statistical analyses were performed with Stata version 15 (StataCorp 2019).

## Results

Table 1 presents the characteristics of the study population, stratified by smoking status. Overall, most respondents were 15 years old (45.5%), had no migration background (76.3%), had at least one parent with a high educational level (49.0%), mostly had no or some smoking friends (85.2%), and non-smoking parents (66.5%). Ever smokers were older, more often had parents with a lower educational level, and more often had best friends and parents who smoke compared to never smokers. Generally, respondents scored low on positive beliefs (0.68 out of 3 on average). Ever smokers scored 0.84, while never smokers scored 0.60.

**Table 1:** Characteristics of the study population, stratified by smoking status. (Smoking inequalities: learning from natural experiments—realist survey, Europe, 2016/17)

	Total	Ever smokers	Never smokers
*N*	10,798	3751	7047
Male (%)	48.9	48.6	49.0
*Age (%)*			
14	32.0	23.3	36.6
15	45.5	47.1	44.6
16	22.5	29.6	18.8
*Migrant background (%)*			
None	76.3	77.3	75.8
One parent	12.3	12.6	12.2
Two parents	11.4	10.1	12.0
*Parental education (%)*			
Low	9.1	12.1	7.6
Middle	30.4	35.5	27.7
High	49.0	42.9	52.3
Unknown	11.4	9.5	12.4
*Best friends that smoke (%)*			
None	43.1	15.7	57.7
Some	42.1	51.7	37.0
Most	13.0	28.1	4.9
All	1.8	4.5	0.4
*Parental smoking status (%)*			
None	66.5	53.8	71.9
One	22.2	27.9	19.3
Two or more	12.1	18.3	8.8
*Country (%)*			
Ireland	16.0	11.0	18.6
Finland	14.9	11.7	16.7
The Netherlands	15.8	14.1	16.6
Belgium	13.7	18.3	11.2
Italy	16.3	24.9	11.7
Germany	10.0	7.3	11.4
Portugal	13.3	12.6	13.7
Average positive beliefs score	0.68	0.83	0.60

Table 2 presents the visibility of smoking in the different public and private spaces, stratified by smoking status. Regarding public spaces, respondents most often reported observing smoking at train or bus stations (83.7%) and least often at leisure/sports facilities (35.4%). These findings were similar between countries (see Online Resource 1). As for private spaces, 35.8% and 43.4% had seen others smoke at home or at a friend’s home, respectively. Overall, 95.1% had seen others smoke in at least one public space in the last 6 months, while 56.4% had seen others smoke in at least one private space. We found large differences between ever and never smokers in terms of seeing smoking in private spaces; 77.6% of ever smokers compared to 45.1% of never smokers reported seeing smokers in at least one private space. In public spaces, these differences were much smaller; 97.4% of ever smokers and 93.8% of never smokers reported seeing smokers in at least one public space.

**Table 2:** Visibility of smoking in different locations, stratified by smoking status. (Smoking inequalities: learning from natural experiments–realist survey, Europe, 2016/17)

Smoking visibility (%)	Total	Ever smokers	Never smokers
*At home*			
Yes	35.8	50.0	28.3
No	62.9	48.3	70.6
Never goes here	1.3	1.7	1.1
*At a friend’s home*			
Yes	43.4	66.7	31.0
No	53.9	31.5	65.8
Never goes here	2.7	1.8	3.2
*At a bar or club*			
Yes	59.3	73.2	52.0
No	14.2	10.6	16.1
Never goes here	26.5	16.2	31.9
*At restaurants*			
Yes	55.4	58.4	53.7
No	41.0	38.1	42.6
Never goes here	3.6	3.5	3.5
*At a train or bus station*			
Yes	83.7	87.0	83.4
No	9.3	8.0	10.0
Never goes here	6.0	5.0	6.6
*At leisure/sports facilities*			
Yes	35.4	39.0	33.4
No	55.6	50.8	58.2
Never goes here	9.0	10.2	8.4
*At least one private space*^a^			
Yes	56.4	77.6	45.1
No	43.6	22.4	54.9
*At least one public space*^b^			
Yes	95.1	97.4	93.8
No	4.9	2.6	6.2

^a^Private spaces include home and friend’s home

^b^Public spaces include a bar or club, restaurants, a train or bus station, and leisure/sports facilities

Table 3 presents associations between visibility of smoking in the different public and private spaces and positive beliefs about smoking. Controlled for socio-demographics and country (Model 1), smoking visibility was associated with a higher positive beliefs score. For example, observing others smoke in friends’ homes was associated with a 0.13 point (95% CI 0.11; 0.15) higher score on the 0–3 scale for positive beliefs. After adjusting for smoking status of respondents, friends, and parents (Model 2), the associations for own home (*β *= 0.01, 95% CI − 0.01; 0.04) became non-significant, and friends’ homes (*β *= 0.03, 95% CI 0.01; 0.05) became weaker. In the fully adjusted model (Model 3), respondents who had observed others smoke at restaurants (*β *= 0.03, 95% CI 0.01; 0.05), train or bus stations (*β *= 0.05, 95% CI 0.01; 0.08), and leisure/sports facilities (*β *= 0.04, 95% CI 0.01; 0.06) had a higher positive beliefs score than those who had not observed others smoke at those three locations. Also, respondents who never visited bars or clubs had significantly lower positive beliefs scores compared to those who had visited a bar in the last 6 months, but had not seen others smoke there (*β *= − 0.07, 95% CI − 0.10; − 0.04).

**Table 3:** Associations between the visibility of smoking in different spaces and the positive beliefs score, for the total study population. (Smoking inequalities: learning from natural experiments—realist survey, Europe, 2016/17)

Smoking visibility	Average positive beliefs score	Associations between visibility of smoking and positive beliefs score *β* (95% CI)			
		Model 0^a^	Model 1^b^	Model 2^c^	Model 3^d,e^
*At home*					
No	0.65	Ref	Ref	Ref	Ref
Yes	0.72	**0.07 (0.05; 0.09)**	**0.07 (0.05; 0.09)**	0.01 (− 0.01; 0.04)	0.01 (− 0.02; 0.03)
*At a friend’s home*					
No	0.62	Ref	Ref	Ref	Ref
Yes	0.76	**0.12 (0.10; 0.15)**	**0.13 (0.11; 0.15)**	**0.03 (0.01; 0.05)**	0.02 (− 0.01; 0.04)
*At a bar or club*					
Never goes here	0.54	**− 0.08 (− 0.11; − 0.05)**	**− 0.08 (− 0.11; − 0.04)**	**− 0.06 (− 0.09; − 0.03)**	**− 0.07 (− 0.10; − 0.04)**
No	0.64	Ref	Ref	Ref	Ref
Yes	0.75	**0.10 (0.07; 0.13)**	**0.09 (0.06; 0.12)**	**0.04 (0.01; 0.07)**	0.02 (− 0.01; 0.05)
*At restaurants*					
No	0.64	Ref	Ref	Ref	Ref
Yes	0.71	**0.06 (0.04; 0.08)**	**0.06 (0.04; 0.08)**	**0.04 (0.02; 0.07)**	**0.03 (0.01; 0.05)**
*At a train or bus station*					
No	0.63	Ref	Ref	Ref	Ref
Yes	0.69	**0.08 (0.04; 0.11)**	**0.09 (0.05; 0.12)**	**0.07 (0.03; 0.10)**	**0.05 (0.01; 0.08)**
*At leisure/sports facilities*					
Never goes here	0.67	0.02 (− 0.01; 0.06)	0.03 (− 0.01; 0.06)	0.02 (− 0.02; 0.05)	0.03 (− 0.01; 0.07)
No	0.66	Ref	Ref	Ref	Ref
Yes	0.72	**0.08 (0.06; 0.10)**	**0.08 (0.05; 0.10)**	**0.05 (0.03; 0.07)**	**0.04 (0.01; 0.06)**

Associations presented in bold are statistically significant at the 95% confidence level (*p* < 0.05)

^a^Model 0: Unadjusted model

^b^Model 1: Controlled for age, gender, migrant background, parental education, and country

^c^Model 2: Same as Model 1, plus the smoking status of the respondents, best friends, and parents

^d^Model 3: Same as Model 2, plus the reported visibility of smoking in the five other locations

^e^The intraclass correlation coefficient of the fully adjusted model is 0.007

Table 4 presents the stratified results by smoking status. Magnitudes of most Betas were similar for ever smokers and never smokers. Both ever smokers and never smokers who had observed others smoke at a train or bus station (*β *= 0.07, 95% CI 0.1; 0.13 for ever smokers; *β *= 0.04, 95% CI 0.0; 0.08 for never smokers) and leisure/sports facilities (*β *= 0.05, 95% CI 0.2; 0.09 for ever smokers; *β *= 0.03, 95% CI 0.0; 0.05 for never smokers) had higher positive beliefs scores compared to those who had not seen others smoke in those places. In addition, among never smokers, those who never went to a bar or club had lower positive beliefs scores compared to those who went but had not seen others smoke at bars/clubs (*β *= − 0.08, 95% CI − 0.12; − 0.04).

**Table 4:** Associations between the visibility of smoking in different spaces and the positive beliefs score, stratified by smoking status. (Smoking inequalities: learning from natural experiments—realist survey, Europe, 2016/17)

Smoking visibility	Ever smokers		Never smokers	
	Average positive beliefs score	*β* (95% CI)^a^	Average positive beliefs score	*β* (95% CI)^a^
*At home*				
No	0.83	Ref	0.59	Ref
Yes	0.83	− 0.01 (− 0.05; 0.03)	0.62	0.01 (− 0.02; 0.05)
*At a friend’s home*				
No	0.80	Ref	0.58	Ref
Yes	0.85	− 0.01 (− 0.05; 0.03)	0.65	0.02 (− 0.00; 0.05)
*At a bar or club*				
Never goes here	0.74	− 0.04 (− 0.10; 0.03)	0.49	**−0.08 (−0.12; −0.04)**
No	0.80	Ref	0.58	Ref
Yes	0.85	0.01 (− 0.05; 0.07)	0.67	0.03 (− 0.01; 0.07)
*At restaurants*				
No	0.80	Ref	0.56	Ref
Yes	0.85	0.02 (− 0.02; 0.06)	0.63	0.03 (− 0.00; 0.05)
*At a train or bus station*				
No	0.79	Ref	0.57	Ref
Yes	0.83	**0.07 (0.01; 0.13)**	0.60	**0.04 (0.00; 0.08)**
*At leisure/sports facilities*				
Never goes here	0.83	0.05 (− 0.01; 0.11)	0.57	0.02 (− 0.02; 0.07)
No	0.80	Ref	0.59	Ref
Yes	0.87	**0.05 (0.02; 0.09)**	0.63	**0.03 (0.00; 0.05)**

Associations presented in bold are statistically significant at the 95% confidence level (*p* < 0.05)

^a^Controlled for age, gender, migrant background, parental education, best friends that smoke, parental smoking status, country and the reported visibility of smoking in the five other locations

## Discussion

### Key findings

Almost all respondents reported observing others smoke in at least one public space (mostly at train/bus stations), while 56% reported smoking visibility in at least one private space. Adolescents who had observed others smoke at restaurants, train or bus stations, and leisure/sports facilities had more positive beliefs about smoking than those who had not observed smoking in those spaces, even after controlling for best friends’ and parents’ smoking status and the visibility of smoking in the remaining spaces. Associations were of similar magnitude for both ever smokers and never smokers.

### Strengths and limitations

This is the first study that investigated the relationship between the visibility of smoking in different spaces and positive beliefs about smoking. In comparison with other international surveys, such as the ESPAD and HBSC, the SILNE-R survey includes more detailed measurements on the visibility of smoking and thus can be considered an advancement that substantially contributes to the existing literature. Also, the large international sample of European adolescents provides estimates that are more widely generalizable than single-country studies.

Some limitations of the present study should be taken into account. First, the cross-sectional design of this study does not allow inferences about causality. It is conceivable that the visibility of smoking in public spaces influences the positive beliefs that adolescents have about smoking, but it is also possible that adolescents with positive beliefs about smoking are more likely to go to places where people smoke.

Secondly, the self-reported, retrospective nature of the survey may have resulted in recall bias as respondents may have inaccurately recalled whether they had seen people smoke in different locations within the last 6 months. If those adolescents who are more positive about smoking are more likely to recall this information correctly, for example because they are more aware of the smokers in their surroundings, this may account for part of the association.

Thirdly, respondents could not specify in the survey how often they had seen others smoke in a certain location within the last 6 months. It is possible that an increased frequency of exposure to others smokers leads to more positive beliefs about smoking.

### Interpretation of the findings

The visibility of smoking in private spaces was relatively low among never smokers, possibly because they are less likely to have friends and parents who smoke (Alves et al. 2016). However, the visibility of smoking was high in public spaces among both never smokers and ever smokers. A possible explanation for the high visibility of smoking in public spaces is that European countries lack smoking bans in most outdoor spaces (Martínez et al. 2014). To our knowledge, while European countries have complete smoking bans inside leisure/sports venues and railway stations, they rarely prohibit smoking outdoors (e.g. at bus stops, open-air train platforms, surroundings of football pitches and sports facilities, playgrounds, and in parks). In addition, train stations and bus stops are typically spaces where people need to wait, which may stimulate smokers to smoke more in those spaces (Shiffman et al. 2002). Our findings underline the importance of extending smoking bans to outdoor spaces, in line with FCTC Article 8 (World Health Organization 2007).

Smoking by others was observed by a large majority (80.7%) of adolescents who had visited a bar or club in the last 6 months and 57.4% of adolescents who had visited a restaurant within the last 6 months. These high percentages are contrary to findings from the 2017 Eurobarometer report, according to which only 18.0% of adults who had visited a bar and 5.7% of adults who had visited a restaurant within the last 6 months, in the same seven countries that were included in our study, reported seeing people smoke inside those establishments (TNS Opinion & Social 2017). There are two possible explanations for the higher visibility reported by our respondents. First, it is possible that adolescents go to different bars/clubs (e.g. teen nightclubs) and restaurants (e.g. fast food joints) compared to adults and that the visibility of smoking is higher in those establishments. Second, our survey did not explicitly distinguish between smoking inside or just outside bars/clubs and restaurants. Smokers who are not allowed to smoke inside a bar, club, or restaurant often relocate their smoking outdoors (Rooke et al. 2013; Kennedy et al. 2012), so it is possible that the high percentages found in our study are a reflection of the visibility of smoking around bars and restaurants rather than inside. This explanation is supported by the finding that 71.5% of Irish adolescents reported seeing others smoke at a bar or club (see Online Resource 1), while Ireland has comprehensive indoor smoking legislation (Joossens and Raw 2017).

We found that the association between positive beliefs and observing others smoke in private spaces was mainly attributable to the smoking status of respondents, friends, and parents, confirming our hypothesis that role models such as peers and parents influence the smoking beliefs of adolescents. Interestingly, we found associations for public spaces after adjusting for friends and parents who smoke, and therefore the role of others in public spaces may not be negligible. While friends and parents may be important role models in the home environment, other role models can be present in public spaces, such as leisure/sports facilities. These role models may include older peers and sports teachers who can influence adolescents’ smoking beliefs and behaviour (Escario and Wilkinson 2018; Poulsen et al. 2002). We also found an association for observing others smoke at train or bus stations. It is possible that even observing strangers smoke in everyday-life contexts where smoking is not expected or socially accepted shapes youth perceptions of smoking (Alesci et al. 2003).

The visibility of smoking in bars was not associated with positive beliefs about smoking. Smoking in bars and clubs is seen as normal, socially acceptable behaviour (Nichter et al. 2010; Rooke et al. 2013), meaning that the visibility of smoking may not further influence the beliefs of youth who go out. However, we found that never going to bars was associated with a lower positive beliefs score. As smoking is strongly associated with going out, there may be a selection effect in which adolescents who are less positive about smoking are less likely to visit bars. However, there could also be a causal influence in that youth who do not go out have never been exposed to a smoking culture that shapes positive beliefs about smoking. Other studies suggested smoking bans in bars, and their direct surroundings can play a role in changing the smoking culture (Hamilton et al. 2007; Ritchie et al. 2010), thus preventing adolescents from developing positive beliefs about smoking in the long run.

The relationships we found between the visibility of smoking and positive beliefs among never smokers were as expected, but we also found substantial associations among ever smokers. This suggests that ever smokers may also be susceptible to the visibility of smoking in public spaces. While more positive beliefs among never smokers can lead to smoking initiation (Song et al. 2009), more positive beliefs among smokers can make it more difficult for them to quit smoking (Kahler et al. 2007), indicating the importance of addressing these beliefs among both never and ever smokers.

Finally, it is important to note that the observed effect sizes were relatively small, suggesting that the visibility of smoking possibly does influence adolescents’ positive beliefs about smoking, but that other factors such as the smoking statuses of the individual, friends and parents, tobacco marketing, TAPS, and tobacco policies may have a greater effect on positive beliefs about smoking. However, reducing the visibility of smoking does affect more than youth beliefs of smoking. A reduced visibility of smoking in public spaces may contribute to a perceived social unacceptability of smoking (Alesci et al. 2003; Albers et al. 2004), which may lead to an increase in voluntary home smoking restrictions (Mons et al. 2013). Ultimately, the denormalization of smoking will have an important societal role in long-term prevention of smoking initiation (Zaleski and Aloise-Young 2013) and smoking cessation (Myers and MacPherson 2008).

### Conclusion

Most participants reported observing others smoke in public spaces, especially at train/bus stations, bars/clubs, and restaurants. While positive smoking beliefs in the home environment were explained by the smoking status of friends and parents, observing others smoke in different public spaces was found to be associated with more positive beliefs about smoking. Given the high adolescent-reported visibility of smoking in public spaces and associated positive beliefs about smoking, the implementation of more comprehensive smoking bans in public spaces where minors are present may be needed to prevent adolescents from developing positive beliefs about smoking. This will likely also contribute to the denormalization and prevention of smoking among youth in European countries.

## Electronic supplementary material

Below is the link to the electronic supplementary material.

Supplementary material 1 (PDF 103 kb)
