# Supplementary material for: The visibility of smoking in Europe and its relationship with youth’s positive beliefs about smoking
Source: Int J Public Health. 2019 Sep 5;64(9):1335–44. doi: 10.1007/s00038-019-01288-z (PMC6868106; doi:10.1007/s00038-019-01288-z)
Supplement: Supplementary file 1 — Supplementary material 1 (PDF 103 kb) [file 38_2019_1288_MOESM1_ESM.pdf]

Journal: International Journal of Public Health

Article title: The visibility of smoking in Europe and its relationship with youth's positive beliefs about smoking

Supplementary table 1: Visibility of smoking in different locations, stratified by country. (Smoking Inequalities: Learning from Natural Experiments - Realist survey, Europe, 2016/17)

Smoking visibility (%)	Ireland	Finland	Netherlands	Belgium	Italy	Germany	Portugal
**At home**							
Yes	28.9	23.9	29.8	47.6	47.6	44.0	31.9
No	70.2	72.7	69.7	49.8	51.7	55.1	67.8
Never goes here	0.9	3.4	0.5	2.6	0.7	0.9	0.3
**At a friend's home**							
Yes	36.3	25.5	40.6	63.2	62.7	37.1	36.0
No	61.9	70.2	57.6	33.4	35.7	58.4	61.5
Never goes here	1.8	4.3	1.8	3.5	1.6	4.6	2.5
**At a bar or club**							
Yes	71.5	22.7	51.3	47.8	87.8	40.2	86.6
No	16.7	34.2	13.4	10.9	6.5	13.1	3.1
Never goes here	11.8	43.1	35.3	41.3	5.7	46.7	10.3
**At restaurants**							
Yes	49.4	25.8	68.9	46.1	65.1	63.8	70.6
No	49.2	66.7	28.2	49.1	31.7	5.6	28.7
Never goes here	1.3	7.5	2.9	4.8	3.2	30.7	0.7
**At a train or bus station**							
Yes	84.5	84.7	83.7	96.6	74.1	94.2	79.5
No	12.4	10.9	6.5	1.2	14.0	3.3	14.2
Never goes here	3.1	4.4	9.8	2.2	11.9	2.5	6.3
**At leisure/sports facilities**							
Yes	21.5	29.3	48.9	26.4	39.0	36.8	46.6
No	72.2	64.1	45.2	59.0	49.0	53.3	44.9
Never goes here	6.3	6.6	5.9	14.6	12.0	9.9	8.5
